# The Feline and the Swine: A Peculiar Case in an Allergy Clinic

**DOI:** 10.7759/cureus.46284

**Published:** 2023-09-30

**Authors:** Mahlon R Kile, Matthew D Lucas, Malika P Ganguli, Ves Dimov

**Affiliations:** 1 Internal Medicine, Ross University School of Medicine, Bridgetown, BRB; 2 Internal Medicine, American University of the Caribbean School of Medicine, Cupecoy, SXM; 3 Medicine, Ross University School of Medicine, Bridgetown, BRB; 4 Allergy and Immunology, Cleveland Clinic Hospital of Florida, Weston, USA

**Keywords:** cross-reactivity, cross-sensitization, albumin sensitivity, meat allergy, pork cat syndrome

## Abstract

Cross-reactivity between mammalian proteins, such as that in Pork Cat Syndrome, remains a topic of great interest. This syndrome, characterized by an immunoglobulin E (IgE)-mediated response to porcine albumin triggered by sensitization through cat epithelium, has been sparsely documented. We discuss a 41-year-old female who developed a pruritic rash within 30 minutes of consuming pork. Notably, she exhibited elevated serum IgE levels with specific reactions to cat dander, dog dander, and pork. A skin prick test for pork was positive. The patient was treated conservatively with allergen avoidance, vitamin D supplementation, fexofenadine, and doxycycline for systemic reactions, and topical corticosteroids for localized skin reactions, yielding a resolution of symptoms. This case underscores the significance of recognizing rare cross-reactivities in allergy and immunology and the manifestations of Pork Cat Syndrome, necessitating a comprehensive patient history and awareness for improved diagnosis and management.

## Introduction

Cross-reactivity between mammalian proteins has always intrigued allergists and immunologists. One such interesting phenomenon is the molecular mimicry between certain proteins of different animal species, leading to unexpected allergic responses. Beyond well-known cross-reactivities like those between birch pollen and apple or between latex and bananas, mammals have their share in the debate. The molecular structure of serum albumins, which are highly conserved across many animals of shared lineage, provides a backdrop for such cross-reactions. For instance, individuals allergic to α-1,3-galactose, a carbohydrate found in the meat of some mammals, may react to red meats such as beef, lamb, and pork [1,2]. Similarly, milk proteins, particularly caseins and whey proteins, have shown cross-reactivity with beef proteins, explaining some beef allergies in milk-allergic individuals [3]. Among these cross-reactivities, Pork Cat Syndrome is a less commonly encountered, yet intriguing, topic. This condition involves an immunoglobulin E (IgE)-mediated immune reaction to porcine albumin, initiated by sensitization through cat epithelium, generally manifesting post-adolescence [4]. Drouet et al. first described this interesting interaction in 1994 [5], and few cases are reported sporadically. Not unlike other allergies, the presentation of this condition is notably diverse, with patients displaying symptoms ranging from urticaria to anaphylaxis [6-9]. Data on the incidence of those with cat allergies who develop pork allergy indicate it ranges from 1-3% [10]. Here, we present a case of suspected Pork Cat Syndrome.

## Case presentation

A 41-year-old female with a past medical history of chronic allergic rhinitis and hypothyroidism presented at our clinic post-exposure to pork, which led to a pruritic rash developing within 30 minutes of consumption. This dermatitis was characterized by a widespread eczematous pruritic rash on both upper and lower extremities, intensified on her fingers, accompanied by sporadic episodes of urticaria. Serum IgE levels were elevated at 220.6 kU/I, and she exhibited a class 2 reaction to specific allergens like cat dander (IgE 0.75 kU/I), dog dander (IgE 2.4 kU/I), and pork (IgE 1.17 kU/I). Laboratory tests pinpointed additional vitamin D deficiency. A patch test for contact allergens came back negative, and a skin prick test for pork displayed a positive reaction. Comprehensive lab values gathered for serum tryptase, Helicobacter (H.) pylori, α 1 and 2 globulin, gamma globulin, hepatitis C, complements 3 and 4, complement 1 esterase inhibitor, and urticaria-inducing activity were all within normal limits. Our treatment approach encompassed vitamin D supplementation, fexofenadine, doxycycline, and allergen avoidance. Over-the-counter occlusive agents and topical steroids were recommended. While urticaria subsided, sporadic pruritus remained. With the underlying laboratory cause of this pruritus remaining elusive, our suspicions were directed toward Pork Cat Syndrome as the culprit behind this pruritus.

## Discussion

The pathogenesis of Pork Cat Syndrome involves the sensitization to feline epithelium, subsequently leading to cross-reactivity with other porcine albumin. However, presentation is variable and treatment remains in its infancy, necessitating further research [10]. While documented cases of Pork Cat Syndrome in published and clinical science remain sporadic, they contribute to the growing body of literature exploring allergic responses prompted by sensitization through unique animal sources. Accompanying this growth are the various symptomatic manifestations of those affected.

Alvarez-Perea et al. chronicled a case where Pork Cat Syndrome manifested as rhinoconjunctivitis alongside symptoms like coughing, wheezing, and shortness of breath. Initially diagnosed with pollen and cat dander-induced allergic rhinoconjunctivitis and mild intermittent asthma at age 14, the patient's condition heightened to new levels when she undertook a job involving the handling of cured meats and cutting of pork bones at age 20. Notably, while prior to this employment her symptoms were manageable with cat avoidance and seasonally manifested, the new occupation saw a surge in rhinoconjunctivitis and recurrent asthma exacerbations. A nasal challenge with a pork bone extract confirmed the diagnosis when it resulted in intense rhinorrhea, sneezing, and a 52% reduction in peak nasal inspiratory flow. This patient’s symptoms were effectively controlled with oral corticosteroids.

In contrast, Posthumus et al. described a 21-year-old female presenting with abdominal cramping, nausea, and oropharyngeal itching beginning after pork consumption. These symptoms were also accompanied by rhinitis, conjunctivitis, and urticaria. Immunoassays displayed not only elevated total IgE but also reactions to both cat dander and pork allergens. Confirmatory laboratory tests and presenting symptoms led to the conclusion of Pork Cat Syndrome. This patient was advised to restrict pork consumption.

These cases cast a spotlight on the diverse symptomatology related to Pork Cat Syndrome - be it gastrointestinal, respiratory, or dermatological in nature. Accordingly, the present case serves as yet another example of the diversity of allergic presentations with the dermatological symptoms of rash and persistent pruritus prevailing. It is crucial to note an anaphylactic response has been recorded and remains yet another manifestation requiring immediate intervention [8,9]. With the treatment regimen in this case encompassing allergen and diet avoidance, vitamin supplementation, and antihistamines, and with knowledge of the diverse manifestations of Pork Cat Syndrome, this report underscores the essence of a comprehensive consideration of diagnoses and management approach for rare Pork Cat Syndrome patients, and further considerations should be made regarding available treatment options, such as that suggested in Barradas Lopes et al., with specific Fel d 2 immunotherapy [10].

## Conclusions

Through this case, we accentuate the importance of recognizing and understanding rare instances like Pork Cat Syndrome in allergy and immunology. Such unique cross-reactivities call for a continued dedication to acquiring a detailed and comprehensive patient history, along with an awareness of Pork Cat Syndrome, ultimately leading to improved diagnosis and management. In an effort to improve the quality of life of these patients, this case dictates a relevance that future management of these patients should involve education among those with allergies to cat epithelium, and their own risk of possible cross-sensitization. In the event of a cross-sensitized reaction, multiple reports depict antihistamines, corticosteroids, and anaphylaxis protocols being utilized as the first-line treatment for Pork Cat Syndrome. This case and those before it serve as awareness of the diagnosis itself and the variety of manifestations, and highlight treatment and prevention options for patients with this intriguing condition.
